# Medical students’ situational motivation to participate in simulation based team training is predicted by attitudes to patient safety

**DOI:** 10.1186/s12909-017-0876-5

**Published:** 2017-02-10

**Authors:** Cecilia Escher, Johan Creutzfeldt, Lisbet Meurling, Leif Hedman, Ann Kjellin, Li Felländer-Tsai

**Affiliations:** 1 Department of Clinical Science Intervention and Technology (CLINTEC), Division of Anaesthesia and Intensive Care, Karolinska Institutet, Stockholm, Sweden; 2 Center for Advanced Medical Simulation and Training (CAMST), Karolinska University Hospital, Stockholm, Sweden; 3 Department of Psychology, Umeå University, Umeå, Sweden; 4 Department of Clinical Science Intervention and Technology (CLINTEC), Division of Orthopaedics and Biotechnology, Karolinska Institutet, Stockholm, Sweden; 5 Department of Clinical Science Intervention and Technology (CLINTEC), Division of Surgery, Karolinska Institutet, Stockholm, Sweden

**Keywords:** Simulator, Teamwork, Medical education, Situational motivation, Attitudes, Patient safety, Surgery, Clinical performance, Crew resource management

## Abstract

**Background:**

Patient safety education, as well as the safety climate at clinical rotations, has an impact on students’ attitudes. We explored medical students’ self-reported motivation to participate in simulation-based teamwork training (SBTT), with the hypothesis that high scores in patient safety attitudes would promote motivation to SBTT and that intrinsic motivation would increase after training.

**Methods:**

In a prospective cohort study we explored Swedish medical students’ attitudes to patient safety, their motivation to participate in SBTT and how motivation was affected by the training. The setting was an integrated SBTT course during the surgical semester that focused on non-technical skills and safe treatment of surgical emergencies. Data was collected using the Situational Motivation Scale (SIMS) and the Attitudes to Patient Safety Questionnaire (APSQ).

**Results:**

We found a positive correlation between students’ individual patient safety attitudes and self-reported motivation (identified regulation) to participate in SBTT. We also found that intrinsic motivation increased after training. Female students in our study scored higher than males regarding some of the APSQ sub-scores and the entire group scored higher or on par with comparable international samples.

**Conclusion:**

In order to enable safe practice and professionalism in healthcare, students’ engagement in patient safety education is important. Our finding that students’ patient safety attitudes show a positive correlation to motivation and that intrinsic motivation increases after training underpins patient safety climate and integrated teaching of patient safety issues at medical schools in order to help students develop the knowledge, skills and attitudes required for safe practice.

**Electronic supplementary material:**

The online version of this article (doi:10.1186/s12909-017-0876-5) contains supplementary material, which is available to authorized users.

## Background

Patient safety is an unquestionable goal of healthcare and education in the healthcare professions [1]. Although the subject is addressed in medical schools, few have managed to fully integrate the subject into their curriculum. WHO has published an extensive framework to help healthcare educators address safety issues in the curricula of basic education for the healthcare professions [2]. Teamwork skills have been identified as crucial for patient safety and hence an important goal for medical education [3]. One of the recommended educational efforts to enhance patient safety in medical education is through medical simulation [4, 5]. Immersive simulator based teamwork training is costly in terms of time, faculty and material. In order to optimize the effect of simulation-based teamwork training (SBTT) a number of quality features of the training have been identified as important [6, 7]. Also, repetitive SBTT is recommended in order to enhance continuous professional development and patient safety [3].

Students’ motivation is known to be of prime importance for learning but so far little is known about medical students situational motivation regarding SBTT [8, 9]. Motivation for and engagement in SBTT is a major concern in order to optimize the use of this resource. According to Self-Determination Theory [10], individuals can be intrinsically motivated (wanting to learn for learning’s sake) and/or extrinsically motivated (wanting to learn for external rewards). Students who are highly motivated will increase their efforts, raise their goals and perform better.

In recent years attitudes to patient safety have been monitored among healthcare providers, as a measure of the safety climate at a particular workplace or within a profession. Studies have shown correlations between the safety climate and patient outcome [11, 12], as well as staff wellbeing [13]. Medical students’ attitudes to patient safety can be scored as a measure of the safety climate and level of awareness of patient safety issues at medical school [14–18]. Changes in attitudes to patient safety are also used to monitor the effect of interventions and to follow development of safety attitudes during medical school [19, 20]. Studies have shown that interventions such as an e- learning course can improve medical students attitudes to patient safety [21].

SBTT is a valuable but expensive tool in patient safety education. In order to guide educators to improvements of patient safety curricula we were interested in students’ attitudes to patient safety and the development of different kinds of situational motivation in relation to SBTT. The main aims of the present study were to investigate a possible correlation between self-assessed patient safety attitudes and situational motivation and if SBTT motivates to further training.

Our hypotheses were that patient safety attitudes would positively correlate to students’ motivation to participate in SBTT and that intrinsic motivation and identified regulation would increase after training.

## Methods

The study was a prospective cohort study. Ethical approval was obtained from the Regional Ethics Review Board in Stockholm.

### The training

During the 2014 spring semester, 64 medical students were scheduled for SBTT as part of their surgical rotation in the fourth year of medical school. Of these, 56 (88%) – 24 males and 32 females – agreed to participate in the study (Table 1).

**Table 1:** Background data of participating students *n* = 56

Age, mean (range)	28 (22–52) years
Sex	32 Females (57%)
	24 Males (43%)
Healthcare work experience, mean (range)	12 (0–72) months
	Females 16 (0–72) months
	Males 8 (0–48) months
Previous simulation-based teamwork training	39%
Semester	7^th^ semester 27 (48%)
	8^th^ semester 29 (52%)

Timing of the training was chosen in order to integrate SBTT with clinical teaching of surgical emergencies as well as ward rotations were teamwork was addressed. The intended learning outcomes were: effective non-technical teamwork skills derived from the crew recourse management concept as explained in the A-TEAM (All team member scale) program [22] and basic skills in the emergency treatment of critical patients (Additional file 1).

In the beginning of the semester, representatives of the Center for Advanced Medical Simulation and Training gave a lecture covering basic knowledge of non-technical skills and the training goals of the SBTT course. During the entire semester, groups of 3–6 students participated in a compulsory full-day SBTT course, including an introduction, clarification of the learning goals, familiarization with the simulator and the environment, practice on vital signs assessment (ABCDE) and a scenario demonstration. The training included 4–5 pre-programmed and standardized emergency scenarios, each followed by video-enhanced goal-directed debriefing focusing on the A-TEAM teamwork skills and clinical performance. Since the groups needed different amounts of time for the introduction and the scenarios, the number of scenarios differed in accordance to the available time. One student in each scenario was appointed team leader, and an instructor was always present in the scenario to help out with medically related practicalities and provide information about signs the simulator could not display – for example, skin colour. All course instructors were specialized in team training, learning and debriefing, and all clinically active in emergency medicine, anaesthesiology or intensive care medicine. The high-fidelity simulators used were either a Human Patient Simulator (CAE Healthcare, Sarasota, USA) or a SimMan 3G (Laerdal, Stavanger, Norway). Each student participated in 2–4 scenarios and observed his or her peers in 1–2 scenarios. Debriefing was geared to the learning goals, and both peers and instructors provided feedback.

After written consent was obtained, the participants completed the Attitudes to Patient Safety Questionnaire (APSQ) [23] and a questionnaire about their age, previous simulator experience and healthcare work experience. The students completed the Situational Motivation Scale (SIMS) [24] after the introduction and at the end of the training session. A standard course evaluation was filled out at the end of the course.

### Situational Motivation Scale (SIMS)

Situational motivation refers to the motivation individuals experience when they are engaged in an activity [24, 25]. SIMS taps into four types of human motivation as described by Self- Determination Theory [10]. Briefly, intrinsic motivation captures participation in a task out of one’s own will and interest, for its own sake. Identified regulation applies to a task performed as a means to an end and not done for itself; thus a type of extrinsic motivation. Another type of extrinsic motivation is external regulation which occurs when behaviour is regulated by rewards or in order to avoid a negative consequence. Amotivation applies to tasks the aim and purpose of which we do not understand. The students were asked to assess their own motivation for participating in the simulation they were to take part in or had just completed. The version of the scale used was adjusted and translated into Swedish, and the items were rated on 7-point Likert-type scales, with four items covering each type of motivation.

### Attitudes to Patient Safety Questionnaire (APSQ)

The APSQ instrument was developed and validated for medical students [23]. It was used with permission from the authors in its original English version after a pilot test that demonstrated that Swedish medical students found it easy to understand. The instrument includes 26 items rated on 7-point Likert-type scales. Sub-scores include: patient safety training received to date, error reporting confidence, working hours as error cause, error inevitability, professional incompetence as error cause, disclosure responsibility, team functioning, patient’s role in error and importance of patient safety in the curriculum. Scores on each item were added to sub-scores as well as to a total score.

### Post-course questionnaire

At the end of the course day the students filled out a standard post-course questionnaire including questions on aspects of the course, the expected value of the training, and whether they would recommend the course to fellow students. These questions are standard course evaluation questions developed by and used at Karolinska Institutet. The students were asked to state their opinions on the course elements on 6-point Likert-type scales.

### Statistical methods and data management

Statistical comparisons to identify the differences between two independent groups were made by using the Student’s *t*-test for uncorrelated means, after validation for normal distribution using the Shapiro Wilk test, or the Mann–Whitney *U*-test if the normal assumption was violated. In order to evaluate hypotheses of variables in contingency tables, the chi-square test was used or, in the case of small expected frequencies, Fisher’s Exact Test. The Pearson correlation coefficient was used in order to test independence between variables. In addition to that, descriptive statistics were used to characterize the data. All analyses were carried out using the statistical software SAS, version 9.4. The 5% level of significance was considered and in the case of a statistically significant result the probability value (*p*-value) has been given.

## Results

### Situational motivation

Intrinsic motivation and identified regulation improved after training (both *p* < 0.001). External regulation and amotivation correspondingly decreased after training (both *p* < 0.001) (Additional file 2).

### Attitudes to patient safety (APSQ)

The mean total score was 135 (range 106–157) of a maximum score of 182. There were significant differences in the sub-scores disclosure responsibility (*p* < 0.001) and team functioning (*p* = 0.029) related to gender, females scoring numerically higher (Table 2).

**Table 2:** Attitudes to patient safety scores female, male and total mean scores *n* = 56

APSQ-subscore	Female (mean/ SD)	Male (mean/SD)	Total (mean/SD)	*P*-value
Patient safety training received	15.6/2.3	15.0/2.8	15.3/2.5	ns
Error reporting confidence	14.6/2.6	13.7/3.5	14.2/3.0	ns
Working hours as error cause	17.9/3.1	17.5/2.5	17.7/2.8	ns
Error inevitability	17.9/2.0	18.2/1.6	18.0/1.8	ns
Professional incompetence as error cause	18.0/3.4	18.0/2.9	18.0/3.2	ns
Disclosure responsibility	15.5/2.3	12.8/3.1	14.4/2.9	<0.001
Team functioning	13.2/0.9	12.5/1.4	12.9/1.2	0.029
Patient role in reducing error	7.6/3.4	8.4/3.3	7.9/3.3	ns
Importance of patient safety in the curriculum	17.0/2.1	16.0/2.1	16.6/2.1	ns
Total score	137.3/9.7	132.3/11.2	135.16/10.6	ns

In our sample no correlation was found between prior SBTT and APSQ scores. The students found it easy to fill out the questionnaire.

### Relationships between APSQ- and SIMS-scores

**Fig. 1:**
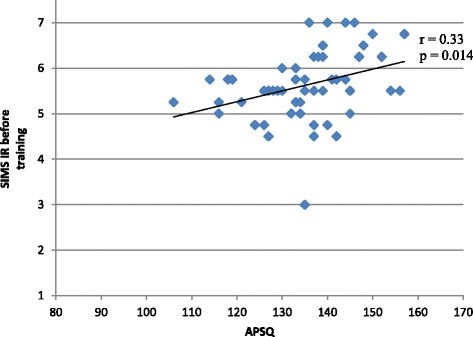
Scores in SIMS – IR (Situational Motivation Scale – Identified Regulation) versus APSQ (Attitudes to Patient Safety Questionnaire) before training

We found correlations between APSQ and SIMS scores. Identified regulation before and after training was positively correlated to total APSQ score (*r* = 0.33, *p* = 0.014 and *r* = 0.40, *p* = 0.002), and amotivation was negatively correlated to total APSQ score, before (*r* = −0.39, *p* = 0.003) and after training (*r* = − 0.32, *p* = 0.017) (Fig. 1).

Intrinsic motivation before training was not correlated to any APSQ score. Identified regulation before training was positively correlated to the sub-scores team functioning (*r* = 0.36, *p* = 0.006), importance of patient safety in the curriculum (*r* = 0.29, *p* = 0.030), working hours as error cause (*r* = 0.31, *p* = 0.022) and total APSQ (*r* = 0.33, *p* = 0.014). Amotivation before training was negatively correlated to disclosure responsibility (*r* = −0.32, *p* = 0.017), team functioning (*r* = −0.53, *p* < 0.001) and total APSQ score (*r* = −0.39, *p* = 0.003).

### Post-course questionnaire

The mean overall judgement of the course was 5.8/6.0. Asking if the students could recommend the course to someone in the same stage of training scored six on a six-point scale from all students (Table 3).

**Table 3:** Post course questionnaire 6-graded Likert like scale *n* = 56

Questions	Mean score
To what extent have you fulfilled the intended learning outcome of the course?	5.2
To what extent did the course build upon your previous knowledge?	5.3
To what extent was the course design appropriate to help you fulfil the learning goals?	5.5
To what extent was the technology (simulator, video) a help to fulfil the goals?	5.6
To what extent did the tutors support your learning?	5.7
To what extent did the course help you reflect on professional attitude?	5.4
To what extent will you have use of what you learned in your future career?	5.6
Which is your overall judgement of the course?	5.8
Would you recommend the course to a fellow student?	6.0
How long do you think this course should be? (hours)	16.6
How often do you think you it would be good for you to participate in SBTT in your future career? (times per year)	3.3

## Discussion

In the present material, including 56 Swedish medical students participating in SBTT with learning goals including emergency treatment and teamwork skills, higher scores on attitudes to patient safety predicted higher identified regulation to participate in SBTT. Further, students’ motivation to participate in additional training increased after the course.

Intrinsic motivation and identified regulation increased significantly after SBTT and amotivation decreased correspondingly. This finding shows that students value the SBTT and are motivated to further team training, a finding that is in line with a study on professional surgical teams [26] and is supported by the high ratings on the post-course questionnaire. No differences in SIMS related to sex were found.

We found that APSQ scores correlated to identified regulation measured before SBTT. However, we did not find a correlation between intrinsic motivation and patient safety attitudes. Effective teamwork when taking care of a critically ill patient is a demanding task in which self-reflection and feedback are important features. According to Self-Determination Theory identified regulation as one type of extrinsic motivation amounting to “doing something because of the good the activity will do”. In this context identified regulation can be considered to be a more important type of situational motivation than intrinsic motivation, which relates to “activities done out of the joy doing them”[25].

Measurements of patient safety attitudes among healthcare staff have been correlated to patient outcome [11, 12]. As safe organizations rely on continuous improvements there is a possibility that motivation to improve and practice are important individual factors contributing to patient safety. Our finding that students’ patient safety attitudes correlated to situational motivation regarding SBTT supports this idea.

In this study attitudes to patient safety were measured using the Attitudes to Patient Safety Questionnaire (APSQ). The questionnaire was chosen because it is validated for students and enables international comparisons of medical schools and may serve as an instrument for monitoring development of the patient safety climate. We found APSQ scores higher or on a par with others’ results using the same instrument [14, 15]. Although the impact of patient safety attitudes at medical school on clinical performance later on has not been established, results from healthcare indicate their importance [11, 12]. The curriculum for patient safety education is one out of many factors likely to have an impact on students’ attitudes. There is evidence that attitudes to patient safety can improve after an intervention [17, 21, 27–30], but another study of medical students did not show changes in attitudes after one episode of SBTT [31]. The safety climate during clinical rotations and also other external contextual factors have the potential to counteract the effect of patient safety teaching [16, 19]. Some studies on patient safety education have showed limited retention of knowledge, skills and attitudes over time, presumably due to a “hidden curriculum” in the safety culture the students experience during their clinical rotation [17]. Given that it is highly interactive, our course has the potential to lead to good retention of knowledge and attitudes, but the extent of this needs further exploration.

Our finding that female students scored significantly higher than males in the APSQ sub-scores disclosure responsibility and team functioning was unexpected but adds to the picture that many factors influence students’ patient safety attitudes. In our study the females longer mean healthcare work experience could explain the gender differences in patient safety attitudes. The implications of the differences in attitudes related to gender and the extent to which differences in attitudes translate to behaviour are not yet fully established and calls for further studies.

The WHO patient safety education initiative recommends practical interactive teaching to achieve the objectives of patient safety education [2]. Simulation and team training are two of the interventions recommended for immediate adoption to enhance patient safety [32]. A SBTT course as described in this study offers an opportunity for students to improve their non-technical and technical skills in providing care in emergencies. To gain knowledge regarding outcome such as improved skills in this particular setting, further studies designed to compare groups are needed. Our findings that patient safety attitudes are correlated to identified regulation before and increased intrinsic motivation after training can be used to optimize timing of SBTT in the curriculum and with respect to other patient safety education. Further it can support the use of distributed SBTT training.

Future studies on the development of patient safety attitudes during undergraduate and postgraduate medical education, impact of different educational interventions and comparison between contexts could further promote the development of patient safety.

### Limitations

The students in our study participated in the SBTT during the entire spring semester of 2014. Due to changes in the curriculum, half of them were in their seventh semester and half in their eighth. This difference in experience could have had an impact on the results. A certain portion of the cohort, 12%, did not participate in the study. At Karolinska Institutet, patient safety teaching is integrated with the curriculum; therefore, we are unaware of the amount of patient safety education the students had received earlier in their training, before our intervention.

## Conclusions

We found that fourth-year medical students at Karolinska Institutet who scored higher in APSQ were more motivated to SBTT. Further, scores on intrinsic motivation and identified regulation increased after training. These findings highlight the importance of early education in patient safety issues and a good safety climate in medical schools. SBTT can enhance students’ motivation for further training and thereby promote continuous development of teamwork skills.

## Additional files

Additional file 1: Training goals, A-TEAM program CRM derived goals for training. (DOCX 30 kb)
Additional file 2: SIMS values, Individual values of the SIMS questionnaire before and after training, the different kinds of motivation calculated. (XLSX 47 kb)

## Abbreviations

A TEAM: All team member scale
APSQ: Attitudes to patient safety questionnaire
CAMST: Center for advanced medical simulation and training
SBTT: Simulator based teamwork training
SIMS: Situational motivation scale.
