# Supplementary material for: Medical students’ situational motivation to participate in simulation based team training is predicted by attitudes to patient safety
Source: BMC Med Educ. 2017 Feb 10;17:37. doi: 10.1186/s12909-017-0876-5 (PMC5301395; doi:10.1186/s12909-017-0876-5)
Supplement: Additional file 1: — Training goals, A-TEAM program CRM derived goals for training. (DOCX 30 kb) [file 12909_2017_876_MOESM1_ESM.docx]

**Training goals:**

Perform A-B-C-D-E

Stabilize vital functions

Call for help

Take a team member role (leader or follower)

Gather information and communicate

Contribute to a shared understanding of the situation

Make collaborative decisions

Coordinate and execute tasks
